# The natural reduction of threat in selected systems of old buildings containing asbestos

**DOI:** 10.1038/s41598-021-04487-y

**Published:** 2022-02-16

**Authors:** Andrzej Obmiński

**Affiliations:** Department: Thermal Physics, Acoustics and Environment, Building Research Institute (ITB), Filtrowa 1, 00-611 Warsaw, Poland

**Keywords:** Environmental sciences, Diseases, Risk factors, Engineering, Materials science

## Abstract

The aim of this study was to measure changes in asbestos dust concentrations over extended use in old building systems. Buildings from different systems during their service lifetimes and after asbestos removal were tested. Asbestos dust concentrations decreased in all building systems due to air exchange and other phenomena in the absence of new active dust sources. Currently, with proper use of those buildings, average values of dust concentrations did not exceed 300–400 f/m^3^ with a downward trend to < 300 f/m^3^. The results of these studies were obtained with a modified optical microscopy technique, verified by SEM and TEM methods. The graphical trends in concentration changes over longer periods illustrate the potency of opposing factors reducing and increasing indoor air pollution. If removal asbestos work was not carried out carefully, single measurements conducted in the short-term and mandated immediately after asbestos removal may not detect hazards that appear over time. Monitoring buildings over longer periods allows detection of significant contamination changes. In many cases, the minimal air contamination undermines the desirability of removing asbestos in old buildings.

## Introduction

### Literature review on differences in asbestos dust concentrations measured in buildings

Some countries such as Germany and France have defined, via internal regulations, acceptable limits regarding the number of asbestos fibers as air pollution in the indoor air of residential buildings after asbestos removal. Other countries have not. Nevertheless, programs for compulsory removal of asbestos from buildings were developed regardless of the level of this pollution. Such a process is expensive, dangerous, and sometimes exacerbates the hygienic condition of the building. In view of the above, to assess the actual need for asbestos disassembly, it is necessary to determine the current state of contamination, as it varies over time and may be subject to constant trends.

These assessments can generate different results, which makes it difficult to interpret and draw conclusions regarding the hygienic condition of the indoor air and the actual need to remove asbestos products, in addition to the real benefits of the planned asbestos disassembly. The assessment process is complicated and can lead to an incorrect analysis, which could pose a risk to the building’s occupants.

According to available data^1,2^, during asbestos removal, asbestos dust concentrations above 1000000 f/m^3^ may be generated in the work zone. This value is 3000–5000 times higher than that generated during normal building use and 1000 times higher than the most frequently recorded values in buildings with poor technical conditions. Individual results of dust concentration measurements at the same places can vary considerably. This applies to the asbestos removal process as well as building conditions before and after asbestos removal^3–10^. Some report low levels of occupational exposure (in relation to PEL) for roofing projects with ACM roofs^11^ (4700–75200 f/m^3^), and in work zones^8^ (600–16000 f/m^3^). The levels of building contamination during use can result^11,12^ in values as low as (70–120 f/m^3^). Dust concentrations reach their highest levels in the workspace, depending on the condition of the products and their destruction^13^, of ~ 50000–388000 f/m^3^ or even^9^ 300000–600000 f/m^3^. Different concentrations, diameters, and sizes of fibers were registered in the same building, e.g. a high concentration of “asbestos structures” 17000 f/m^3^, which may correspond to a low number of respirable fibers (~ 230 f/m^3^) in the same concentration test^14^. Depending on the actions activating the dust in the room^14,15^, the average values in the facility could have increased from 2500 f/m^3^ to 60000 f/m^3^. When measuring using TEM^16,17^ the max. and min. of asbestos dust concentration, the values differed by a factor of 10, as compared to the average concentration of 1500 f/m^3^. Large differences in concentration values for the same room were described^17,18^. It was assumed the elevated level of asbestos fibers in the air of used buildings corresponded to the degree of damage to asbestos products^19–22^. However, damage to the asbestos product was not always sufficient for aerosol formation and many factors contributed to aerosol formation^23–29^. Previous research^25–27^ reports investigating renovated buildings or after renovations confirmed that dust concentrations varied significantly at the same time in single rooms, from 600–7000 f/m^3^. In addition to various short-term changes, pollutants may change in the same premises over longer periods with specific trends. Asbestos dust concentration levels depend on the time elapsed between the moment aerosolized asbestos appears in the air (ACM mounting or removal) to start of sampling (after a long period of building use)^30^. High concentrations of asbestos dust depend on ACM matrix condition and the size of the surface area of asbestos used^31^. Factors which can increase the concentration of dust in the air are also, e.g., renovation inspections, maintenance of equipment, repairs, building upgrades and other activities leading to the disturbance of products. The asbestos content of typical ACM products in the various countries remains at similar levels. These should translate into typical air pollution in typical buildings with typical ACM . Consequently, is assumed correlation between the distribution of asbestos materials and the risk of asbestos dust hazards^31,32^. Elements of construction and location of products, e.g., suspended ceilings and other partitions and their tightness, also have an influence^21,33^. Many features of the building itself , its conditions of use and processes, as gravitational settling, ventilation and attachment to surfaces may modify the value of dust concentrations. Natural factors can act to reduce the concentration of asbestos, which is recorded in normally used buildings^22^ as well as during the monitoring of the demolition process^34–36^. The problem of estimating acceptable dust concentration values and their changes in buildings has been discussed elsewhere widely^37–41^. Short-term changes in asbestos concentrations in an experimental chamber for a period of 30–70 min have been described^22^. Literature reports also monitored the concentration decay after asbestos removal^36^ and evaluation of interior asbestos particle diffusion models based on the Pasquill and Gifford diffusion model for an external environment^33,42^. Changes over decades have not been recorded yet, though computer models have simulated indoor dust concentrations.

### Research goals

The purpose of this study sought to answer several questions: Do dust concentrations decrease over time as a result of e.g. ventilation and dust removal from the building, or do concentrations increase as a result of aging ACM products? Since many decades have passed since buildings with asbestos were constructed and surveyed, and many factors have shaped the level of indoor pollution, can we consider those results (from 1980 to 2015) to be valid today? Is asbestos removal beneficial for all buildings? The originality of this study comes from the use of simplified dust registration over different time periods in the same building, to demonstrate the “self-cleaning” progress of a building over time. This article describes various building categories and use of a common sampling and research method. This ensured constant measurement sensitivity and a repetitive laboratory error. For this reason, this study focused on buildings with potentially significant contaminations so changes in the airborne asbestos dust concentrations could be easily recorded.

## The method of analysis

Indoor air samples from tested buildings were taken periodically, at different time periods, and at approximately similar times of the year (late spring to early autumn). The heating systems were turned off. The rooms in question had similar temperatures and humidity conditions (temperatures 19–24 °C, humidity 40–65%), but had no effect on the concentration of asbestos aerosol in indoor air. (Long-term measurements using the same analytical technique showed that larger differences in these parameters (temperature 10–28 °C, humidity 34–80%) had no effect on aerosol concentrations in indoor air.) However, the effect of wall humidity on the indoor concentration of asbestos aerosol is evident in the described case of Building No. 1. This was due to the drying of the walls after the asbestos was removed from the building. See Fig. 3 and the description of this phenomenon in Chapter 5. This situation represented an extreme renovation and does not occur during use.

Two sampling types (static sampling vis dynamic sampling) were used to determine the proper air sampling technique and the correlation between them, and took into account the sensitivity of the OM technique used in the study. Both sampling types were conducted on the first building, called “BOLETICE”, evidently contaminated with asbestos.

Since the results showed that both sampling methods present a similar mechanism for pollution reduction over 19 years, dynamic sampling was used for the remaining buildings. This made it possible to record small changes in air pollution using a microscopic technique with low OM sensitivity (in relation to electron microscopy techniques).

Static sampling, consisted of air collection under normal, everyday conditions of room use. Dynamic sampling simulated critical usage conditions that might occur in a building. These included impacts against walls, curtains, carpets, chairs, and slamming doors. Airflows from fans were directed towards areas inaccessible to everyday cleaning. These activities were designed to induce re-emission of previously settled dust and fibers not permanently bound to ACM products.

The primary technique in this study was modified OM. Testing of asbestos fiber concentrations in air using OM was conducted by the author as part of accredited testing at the Research Building Institute ITB laboratory (Poland). As a result, the lower cost of OM tests made it possible to perform and compare more of them. Results were verified in selected cases by SEM and TEM analyzes performed in parallel with OM at the same time and place. SEM-EDS studies were performed at the Wessling Laboratory (Germany) according to standard /procedure VDI3492(2013-06)A, BGI/GUV-1505.46(2014-02)A. TEM studies were performed at the EUROFINS Laboratory (France), according to standard NFX43-050 and instruction GAX46-033.

The OM method was modified according to the required asbestos measurement tests adopted for registration of low operational dust concentrations (< 10000 f/m^3^) and the identification of asbestos fibers^43^. The phase contrast method (PCOM) NIOSH 7400^44^, used in this research was suitable only for quantitative analysis^45^. However, this technique facilitates the assessment of asbestos exposure and enables comparison of the results of historical and contemporary research^46^. This technique does not give accurate fiber concentration measurements at the extremely low levels of the air pollution. However, it readily records pollution values 300 f/m^3^ and more. Respirable asbestos fibers were counted using PCOM analogous to the Polish standard PN-88/Z-04202/02 and NIOSH 7400^44^. The prerequisite for counting fibers as asbestos was to record their respirable sizes during phase contrast observation and when the microscope mode was switched to polarized light observation, then to identify the fiber optical characteristics as asbestos. Observations were carried out at a magnification of 500× or 1000× + immersion (if necessary). The modifications to sampling and microscopic analyses for the 7400 NIOSH are reported elsewhere^24,29^.

## Research material: building systems tested

This study focused on typical Eastern European buildings from 1970 to 1990. They differed in construction, size, amount of ACM materials used, structures (outside, inside), cohesion (friable and non-friable), as well as the condition of operation (during use, renovated, and after asbestos removal). The research covered buildings with the so-called “rigid” with brick walls and "non-rigid" construction:

- rigid construction, reinforced concrete construction (insensitive to vibration);
- non-rigid, steel (sensitive to vibration).

**Figure 1:**
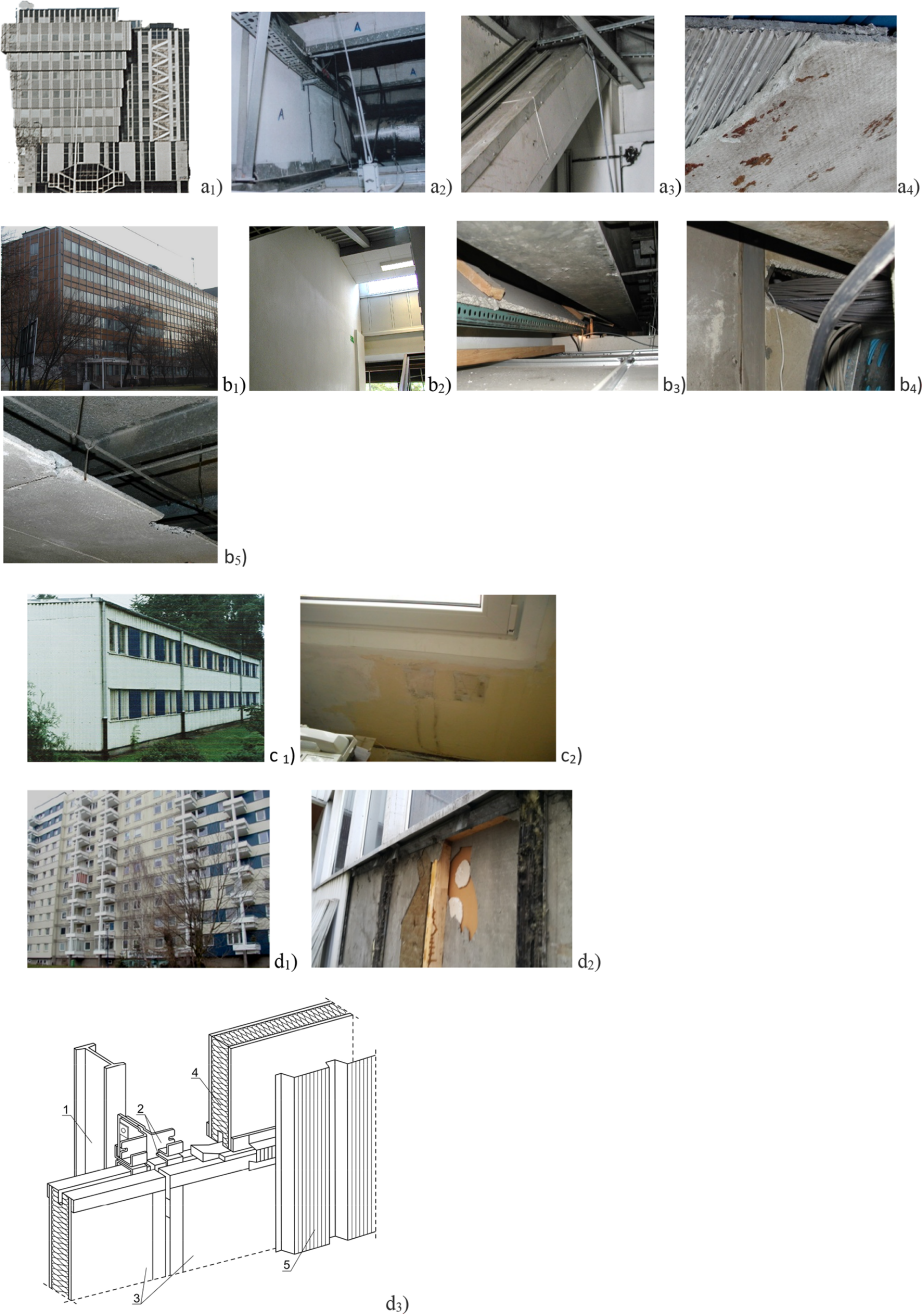
The figures show examples of non-rigid (**a**–**c**) and rigid (**d**) structures. All photographs relate to the buildings and their contamination presented in the article. (**a**_**1**_) View of the “BOLETICE” from CSRS, front sandwich wall with glass facade. Elevation of one of the twin towers of the building; (**a**_**2**_) Fire protection of walls and structures made of “PYRAL” panels—friable ACM (marked "A"), visible from the technical space, above the suspended ceiling of the utility room; (**a**_**3**_) Fire protection of the diagonal structure made of "PYRAL" board; (**a**_**4**_) "PYRAL", a fireproof chrysotile asbestos board used in the “BOLETICE” building. The two layers of the board are bonded with a fine-wave aluminium foil; (**b**_**1**_) “LIPSK” from DDR, building facade. Under the facade there are the “GLAGIT” hard asbestos cement plates contain 13% asbestos; (**b**_**2**_) Internal walls in the stairwell of the building type “LIPSK” contain “SOKALIT”, soft plates (20% asbestos, "friable" ACM as fire—proof product); (**b**_**3**_) Space above the suspended ceiling. The fireproof "SOKALIT" panel is used to shield the electrical cables (in the photo in an oblique position); (**b**_**4**_) Operational damage to "SOKALIT" panels caused by modernization and cabling of the building with a computer network. The picture shows a hole in the panel through which the wiring harness passes; (**b**_**5**_) Damaged "SOKALIT" panels used as a suspended ceiling; (**c**_**1**_) System “BERLIN” from DDR; (**c**_**2**_) Operational damage to the "SOKALIT" panels caused by incorrectly conducted renovation work in the BERLIN building. (Detailed description in the main text). (**d**_**1**_) Different buildings type of “BISTYP” (Polish system): existing as office, hospital and residential on the photograph. (**d**_**2**_) A fragment of the “BISTYP” building wall after removal of the corrugated sheet metal (a facade). Visible layers of PW3/A board to which a cartoon-gypsum board was glued from the inside; (**d**_**3**_) Construction of “BISTYP” sandwich wall with PW3/A or PŻ3W panel. Description of drawing elements: (1) Steel pole, (2) Steel connector, (3) PW3/A or PŻW3/A, sandwich panel with a mineral wool core, faced on both sides with (3)—asbestos cement flat panels, (4) Mineral wool thermal insulation core of PW/3A board, (5) Wall facade made of corrugated sheet steel.

Analyzed systems of buildings are shown in Fig. 1.

## Results

### “BOLETICE” wall systems

**Figure 2:**
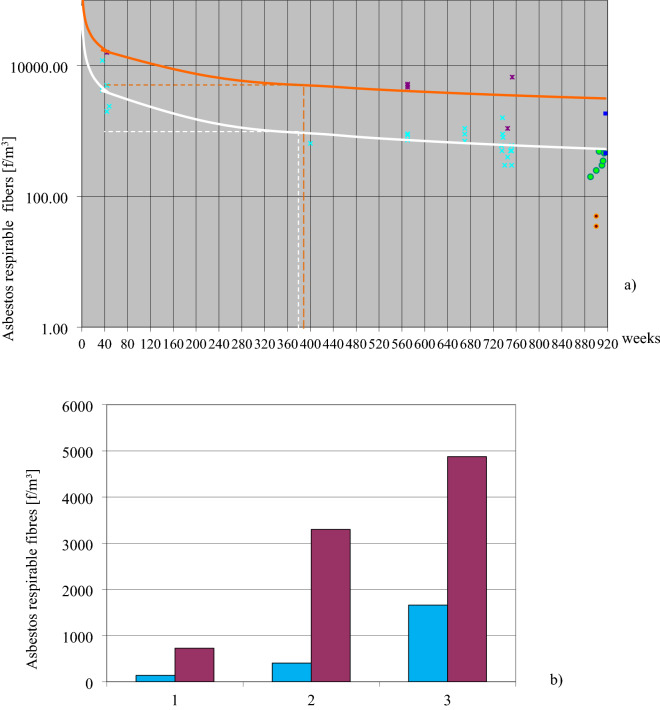
Differences in the asbestos dust concentrations in the air (**a**) The trend of changes in asbestos dust concentrations in the “BOLETICE” system building. Dark purple times: The determination of air samples taken in a dynamic way (one point means the average of two measurements made at the same time and in the same room). Concentration change trends at such samplings were marked as an orange curve. Light blue times: The averaged values of dust concentration obtained during normal building use (standard user activity, working central ventilation, without cabinet fans). Concentration change trends were presented as the white color curve. Measurements made 12 months after the building was no longer in use (after approximately 19 years). The tests were carried out in the same rooms during its operation. Green circle: Test conditions: mechanical ventilation and heating excluded in the building, no operational vibrations related to the use of the building. Dynamic air sampling (analogous to earlier techniques: activities to run dust seated on horizontal surfaces). Blue square: Measurements made at the same time in rooms with fresh, large damage to the sandwich wall created after the building was no longer in use: exposed, damaged, soft products (friable) – “PYRAL” board. Dynamic air sampling: the average asbestos dust concentration was 1200 f./m^3^. Red circle: Measurements made outdoors at ~ 8 m from the building. The mean concentration was < 100 f/m^3^. (**b**) The asbestos dust concentration differences in the air of the three rooms with different levels of dust concentration. Turquoise—static sampling, magenta—dynamic sampling. Approximately two years before the end of the measurements, the building was taken out of use. The overall changes are illustrated by the curve based on average sample values taken statically and dynamically in Fig. 3. This figure shows the same individual 80 measurements, depending on the sampling method, separately and after combining all values into one chart (Fig. 3c). Figures 2 and 3 should be analyzed together because they are shown in the same coordinate system (Y—fiber concentration, X—time in weeks) and present results that complement each other.

**Figure 3:**
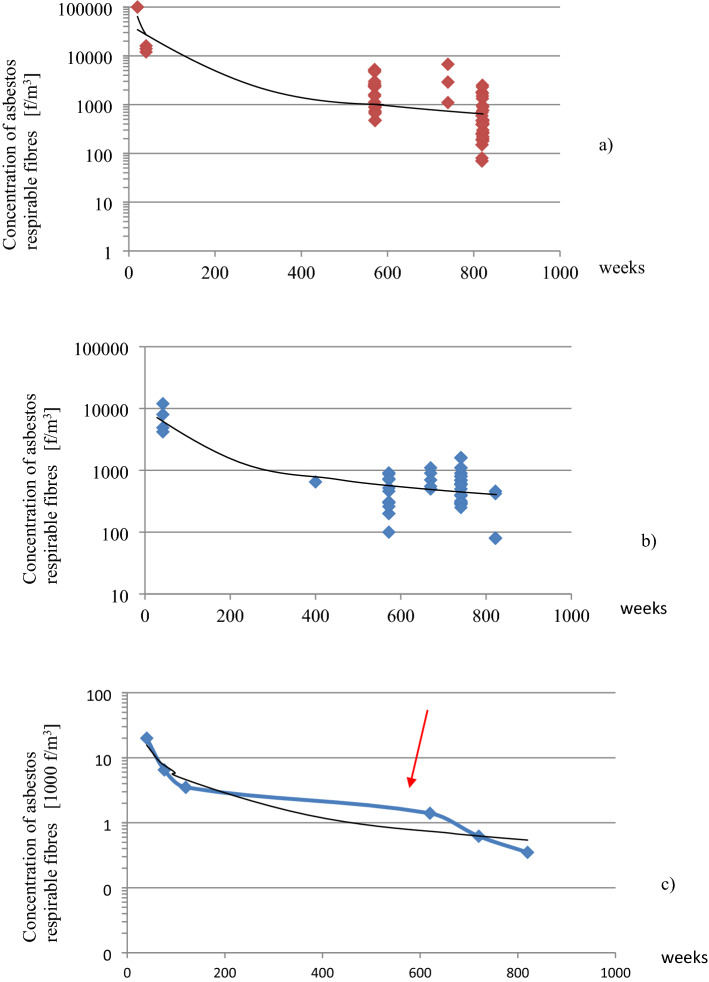
Analysis of changes in asbestos concentration in the BOLETICE building. (**a**) Results of dust concentration in the building during dynamic sampling. (**b**) Results of dust concentration in the building during static sampling. (**c**) Averaged concentration values from static and dynamic sampling in the BOLETICE building as a blue curve. The trend of change is represented by the black curve. The red arrow marks the end of building use.

Measurements were made in this building during its operation from beginning to end of use, 19 years later. Figure 2 shows the values of groups taken from 80 measurements (see Fig. 3a–c). In Fig. 2a those values are marked with colors, and account for the different air sampling methods, static and dynamic. Both curves simulate instantaneous and natural states of use and have been used to analyze trends in dust concentration changes over time. Figure 2b shows differences between values taken from three different rooms.

### “LIPSK”, “BISTYP”,“ BERLIN” systems

**Figure 4:**
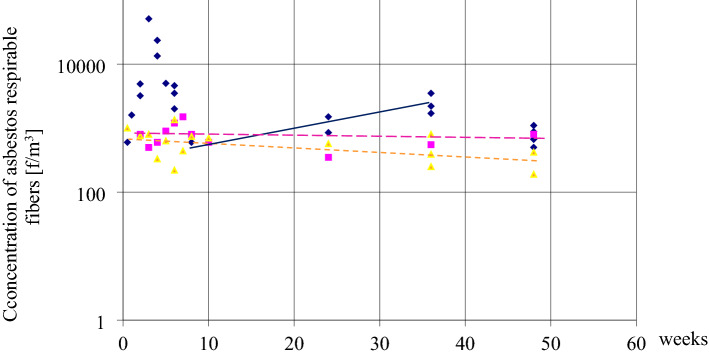
Changes in air pollution of three buildings “LIPSK” within 48 weeks. Dark blue diamond: Building (1) asbestos removal: Changes in asbestos dust concentrations from the commencement of asbestos dismantling, realization, and completion. During removal, dust leaked out of the work zone and increased its concentration during the first three weeks, up to the 5th week, up to 51,000 f/m^3^. The dark blue line indicates an increase in contamination that would not have been detected under the conditions of standard acceptance of the dismantling work and measurements immediately after the completed work. Pink square: Building (2), normal room operation. Yellow triangle: Building (3), normal room operation. After the tenth week of research, special operating rules were introduced in this building (room ventilation, wet mopping of surfaces, daily vacuuming with a HEPA filter, ACM marking, and prohibition of work that violates these products). The dotted orange line indicates a slight downward trend in pollution.

**Figure 5:**
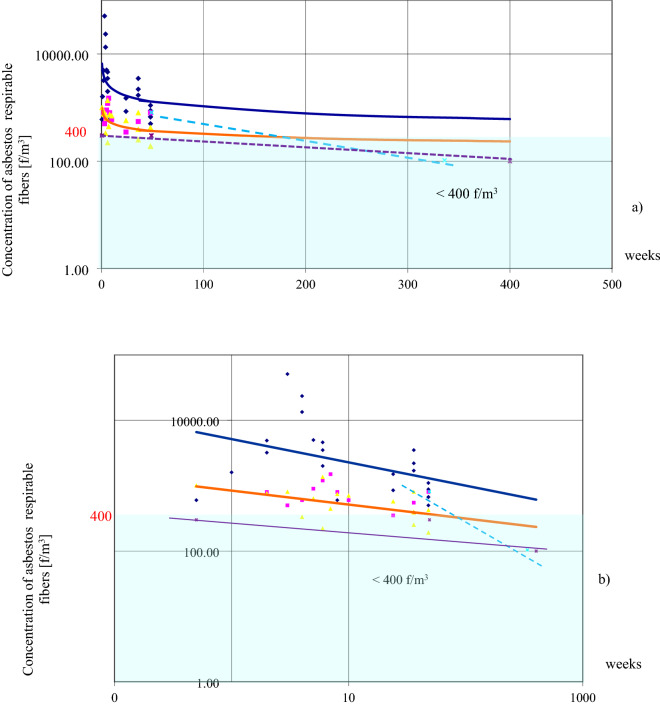
Contamination comparison in rigid (“BISTYP”) and non-rigid structures (“LIPSK”). (**a**) The reduction of the rate of change in the concentration of asbestos dust over a period of time, after reaching a concentration of < 400 f/m^3^. This applies to objects operated in the same way for longer periods of time without damaging the ACM. The vertical axis corresponds to dust concentrations on a logarithmic scale. The horizontal axis denotes the time when measurements were taken (weeks) on a linear scale. 
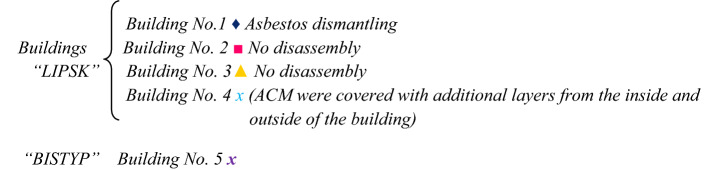
(**b**) The same data as in (**a**). Time of measurements [weeks] on a log scale (on the horizontal axis). The figure shows a steady decline in asbestos concentrations in all cases over an extended period.

Building systems “LIPSK” (non-rigid structures) are presented in Fig. 4 and compared to the “BISTYP” building—(rigid structure) in Fig. 5. Each colored point represents average values from at least six asbestos dust concentration measurements in the individual buildings. There were 40–160 measurements for individual buildings.

In Fig. 4, Building No. 1 has been tested during asbestos removal. Buildings 2 and 3, were in normal use. After 10 weeks of measurements, special operating procedures have been introduced in building 3, which should result in a reduction of pollution and a smaller scattering of results. Statistical analysis in “Statistical analysis” assesses whether these interventions have had a lasting, statistically significant effect.

Figure 5a,b show the detailed results and a contamination comparison between rigid and non-rigid structures. The curves connect measuring points of the same color. They illustrate asbestos dust concentration change tendencies in the tested buildings. Both Fig. 5a,b present the same data with a different coordinate system on the horizontal axis. One of them better illustrates the measured values, the other trends the changes for individual buildings. The rate of asbestos decline achieved in the buildings shown in Fig. 5b clearly differ.

**Figure 6:**
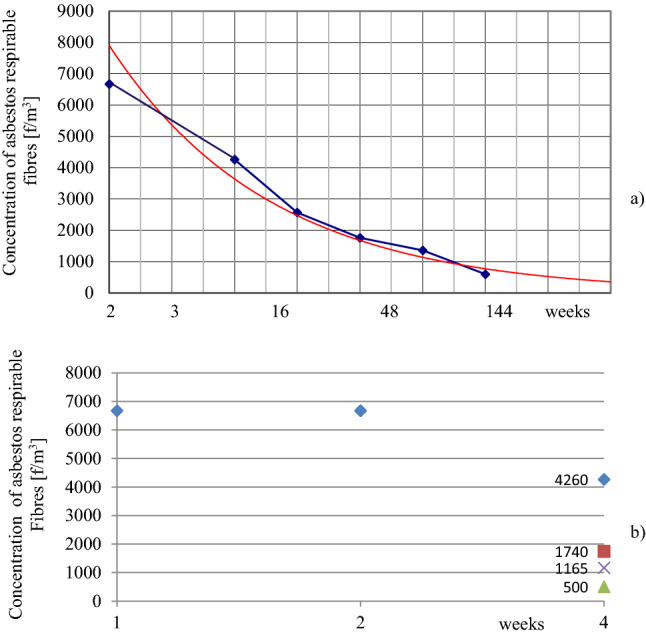
Fiber concentrations in the “BERLIN” building after its interrupted renovation. (**a**) Fiber concentration in the “BERLIN” building during renovation, recorded to the 144th week after the work was stopped (this work was conducted without knowledge of ACM in the building). Average sample values taken on the same day of sampling in different rooms (a time in a logarithmic scale). (**b**) Analysis, including measurements in rooms with increased ventilation and room airing. Green triangle: renovated rooms after 20 days of ventilation. Purple times: rooms not renovated, in use. Brown square: renovated rooms, not in use. Blue diamond: normal use after renovation.

In the case of wrong renovation in a small non-rigid “BERLIN” building, the changes in asbestos dust concentrations recorded in the building in operation we caused by accidentally, unaware disturbing ACM as a result of improperly conducted renovation of walls. It was carried out for several years and then interrupted shortly before the first air tests. (Fig. 6 and Table 1). The red line in Fig. 6 marks the trend line of changes over time.

**Table 1:** The “BERLIN” building, incorrectly renovated without removing asbestos-containing products. The variability of asbestos fibres concentration with time (average values).

Degree of exploitation	Unused rooms (f/m^3^)	Used rooms (f/m^3^)
1 month after renovation	860	3810 (max 6670)
2 months after renovation	–	440^a^ (max 550^a^), 1740–4260
0.5 years after renovation	680	–
1 year after renovation	–	1360–1760
3 years after renovation	–	660–750
Non-renovated used rooms	1420^b^	

^a^Intensive ventilation of rooms between the repair and the beginning of tests.

^b^Active sources of dust contamination present in the rooms, large ACM damage.

The results of measurements in various rooms are shown in Table 1. The disappearance of pollutants formed in indoor air over 144 weeks is shown in Fig. 6a. Four weeks after the work was interrupted, some rooms were intensively ventilated for 20 days. The remaining rooms were used normally, without special ventilation. The results of dust concentration measurements before and after ventilation of similar rooms at the same time are shown in Fig. 6b).

## Discussion of results

### Discussion of tested buildings

#### “BOLETCICE”

According to Fig. 2, the trend in concentration changes could allow estimation of the likely contamination at any time considering different sampling techniques. At any given time, the given point of contamination should not exceed the value of the orange curve and not less than the value of the white curve. For example, in Fig. 2a, at 360 weeks, pollution should range from 1000 to 5000 f/m^3^. The function that optimized the concentrations between periods was determined by a power function curve (y = 54168x^–0.6462^). The approximation of the measured concentration values to time, with the determined curve is high, R^2^ = 0.8341. However, it is not possible to determine the exact concentration value over a certain time interval on this graph because the actual room conditions during sampling are not known. In addition, there are quite large differences in concentrations in different rooms at the same time even for the same sampling technique.

In the first year of operation, maximum of an average value of contamination 16,000 f/m^3^ were recorded using dynamic sampling. The corresponding average value using static sampling was 5800 f/m^3^ (difference 10200 f/m^3^). Between 11 and 15 years of use, the average concentration for dynamic sampling was 3700 f/m^3^ (static, 600 f/m^3^; difference, 3100 f/m^3^). Taking into account the theoretically determined trend that would take place during building operation, between years 18 and 19, the value of dynamic samples would be 3300 f/m^3^ and static samples 500 f/m^3^ (difference 2800 f/m^3^). This change reflects the decreasing difference between static and dynamic sampling over time. It means a disappearance in the difference between “critical” and “normal” use of the building. This was probably due to the successive removal from the premises of dust accumulated in the building during the initial construction period and the dust that settled later not bound by the ACM product matrix. For this reason, there was less discrepancy in the results from static and dynamic sampling (Fig. 3a,b).

A comparison of dust concentrations (see Fig. 3a,b) measured using static and dynamic sampling at the same time indicates that dynamic sampling would have reached the same level as static sampling had it been carried out 5–7 years later. This represents a delay of 5 to 7 years in the indoor air "self-cleaning" process for "critical" building use, compared to "normal" use. Both curves have a similar shape. So, the mechanism for changing the dust concentration in this building is the same for both kinds of fibers (from emission and re-emission), regardless of the concentration size.

The visible effect in Fig. 3c is a drastic decrease in the recorded pollutant values, even from dynamic sampling due to the termination of building use. It means that “kind of use” (or “user type”) is responsible for a significant proportion of the particulate concentrations in a building. Many samples (green points in Fig. 2a, dynamic sampling) taken from a disused building gave an average value of 380 f/m^3^. The difference between the theoretical dynamic trend (orange) during this time and the actual value was 2920 f/m^3^. That indicated the main sources of air pollution depended on the activity of the building’s users and the possibility of activating settled dust. It can be assumed that the higher dust levels recorded by the author in the study of kindergarten and school buildings (confirmed in the literature) are due to above-normal users activity.

#### “LIPSK”, “BISTYP”, “BERLIN”

Examples of these buildings are shown in Figs. 4 and 5.

With reference to Fig. 4 and Building 1, testing was carried out during asbestos removal outside work zone, so contamination values fluctuated widely. We call this contamination “leakage” from the work zones. The maximum of 51000 f/m^3^, was found in Building 1, during the fifth week of removal works. This explains why:

- concentration values fluctuated considerably over a short period of time;
- there were alternating increases and decreases in contamination. (Time was not the determining factor here for the presence of dust in the air. It was the influence of: dismantling of ACMs, cleaning after dismantling, applied technique and diligence of performing the works and cleaning again). Building 1 was compared with Buildings 2, 3, 4 and 5 to show the risks that asbestos removal may pose compared to the operation of different buildings with asbestos and different methods of asbestos control without removal. Asbestos removal and final cleaning were completed after eight weeks. After the dismantling work was completed and the site cleared, slowly increasing dust concentrations were observed. Resumption of use was put on hold. The slow increase in pollution continued from 8 until week 35. This process was caused by the drying of the wet walls. It released of residual fibers left over from the “wet method” of asbestos removal. After week 35 additional cleaning with vacuums and ventilation were re-enforced. This resulted in a decrease in asbestos fiber concentrations to pre-dismantling levels As a result, the contamination decreased to a range of 500–680 f/m^3^. The dynamic changes in contamination over short periods of time were clearly due not to the passage of time but to activities in the building. Over a similar period of time, tests were carried out in two similar buildings No. 2 and 3, which were normally in use.

The concentration drops in Buildings "LIPSK" (No. 1, 2 and 3) have different scales, shapes, different causes and initial and final values. The concentration decrease model for Buildings 4 and 5 differs from Buildings 1, 2, and 3. The small initial values and the small spread of all values (Building 4 and 5) are shown here as straight lines in Fig. 5. This flat trend was likely due to starting the study after higher and variable concentration values had already occurred in the past and dropped over time. The different slopes of their lines stemmed from the different rates of change occurring in buildings 4 and 5.

In all buildings, concentrations of asbestos fibres initially decreased rapidly, then slowed down. This was due to the slower rate of dust removal during ventilation. This in turn was due to the systematically decreasing concentration difference between the indoor and outdoor air. This is a natural consequence of “diluting” a certain amount of dust with a periodic inflow of the same amount of clean air. After the first some years, after maximum pollution, the rapid decrease in the rate of change of dust concentrations gradually decreased asymptotically. After a period of time, change slows down to a range of values < 400 f/m^3^.

This range of values, seen in Fig. 5a,b, was considered by the author to be “low” contamination for these buildings and marked with a light blue area in the graphs. This applies to buildings operated in the same way for an extended period without ACM damage. The measurement results for buildings 2 and 3 show less variation then in case of building 1. In the case of Building 3, the slight trend in dust reduction was due to the implementation of rules for the correct use of the building, which improved air quality. This is explained in the description of Fig. 4 in ““LIPSK”, “BISTYP” ,“ BERLIN” systems”. The statistical analysis in “Statistical analysis” answers the question of whether the changes in these buildings are statistically significant, in particular whether the introduction of the special correction of use in Building 3, results in a significant trend of decreasing concentrations relative to the normal use, observed in Building 2.

A completely different situation is presented by the "BERLIN building" in Fig. 6. The unknowing disturbance of asbestos in this case, during renovation work contaminated the entire building for several years. In selected rooms, intensive air exchange for 20 days without specialist cleaning reduced the contamination eightfold. Negligent asbestos removal companies use this trick instead of the expensive and labor-intensive encapsulation of the building and subsequent decontamination.

#### Statistical analysis

The aim of the analysis was to determine the significance of the changes in dust concentrations obtained following the changes in use of Building 3. (described earlier). These were aimed at reducing and controlling dust in the building. They were introduced after the 10th week of the study. The situation in building 3 was compared with building 2, which was in normal use. The 50-week test period for these buildings was examined separately in three time categories: up to week 10, between weeks 24 and 48 and in total over the whole time interval from week 0 to week 50.

The results of the Pearson’s correlation analysis for Buildings 2 and 3 in hall time of testing showed for:

$${\text{Building 2}}, {\text{p }} = \, 0.{415};{\text{r}} = - 0.{274};{\text{ R}}^{{2}} = \, 0.0{8};$$

$${\text{Building 3}},{\text{ p }} = \, 0.0{89};{\text{ r }} = \, - 0.{439};{\text{ R}}^{{2}} = \, 0.{19};$$

Note:

p is the *p*-value of the test (*test probability).*

p < 0.05 the relationship between elapsed time between tests and dust is statistically significant.

0.05 < *p* < 0.1 this relationship is significant at the level of statistical tendency.

*r* is the correlation coefficient.

Correlation coefficient value |r|:

1. low 0–0.3
2. moderate 0.3–0.5
3. strong 0.5–0.7
4. very strong 0.7–1

R^2^ is the Coefficient of determination.

Statistical analysis over the whole study period showed a low to moderate linear correlation between the passage of time and the decrease in dust concentration. For Building 2 low correlation, insignificant association, no statistical tendency. For Building 3 a statistical trend of decrease. The numerical data, the linear correlation analysis between time and dust concentration and conclusion are presented in Appendix 1.

### Data summary

In the opposite to the thesis of a high risk of pollution in old buildings, this article shows that in many old buildings the risk of asbestos dust has decreased over time. The final trend in the change and the risk of exposure to asbestos dust is determined by the dominance of opposing factors. Some of them lead to an increase in the aerosol and others to its disappearance. The current concentration of asbestos fibers in the indoor air of “rigid structures” such as “BISTYP” is often < 300 f/m^3^. After extended use, when the asbestos-layered walls have been covered with an additional layer (for example, after the building has been thermally insulated, as in the case of Fig. 2d3 and Building No. 4 in Fig. 5), the contamination may even drop below 100 f/m^3^. These values can be called an “insignificant” surplus of asbestos fiber concentration over background values, i.e. atmospheric air.

This questions the advisability of removing asbestos from some building systems; if the present value of asbestos dust in them is low and the work of removing these products temporarily may worsen the existing indoor air quality.

In building structures “rigid”, with sandwich walls containing ACM, the highest probability of the concentration of respirable asbestos fibers (mode) ranges from 0 to 300 f/m^3^. For “non-rigid” construction buildings, which depend on many factors, including the technical condition, this concentration range was significantly wider (0–1000 f/m^3^).

Table 2 shows the collective results of indoor air pollution for asbestos-containing building systems measured in this work.

**Table 2:** Indoor air pollutant concentrations for several typical asbestos building systems. (A—Average value from 20–200 tests of asbestos respirable fiber concentrations in all tested rooms; B—Median of recorded values; C—Mode—the value with the highest probability of occurrence; D—The highest concentration of fibers in the air; E—The lowest concentration of fibers in the air).

No		Building system	A	B	C	D	E	Comments
1	Construction rigid^a^	“BISTYP”	320	420	–	590	0	6 years after the start of operation
2			< 300	< 300	0	< 300	0	36 years after the start of operation
3			0	0	0	0	0	
4			0	0	0	0	0	
5			< 300	0	0	300	0	
6		Other different sandwich walls inside the curtain wall	330	450	< 300	1000	0	~ 40 years from the start of operation
7			< 300	< 300	< 300	600	< 300	
8			< 300	0	0	< 300	0	
9			< 300	< 300	< 300	300	0	
10			< 300	< 300	< 300	340	0	
11	Construction non-rigid^b^	Buildings: type “LIPSK”, with different ACM conditions	710	800	760	1200	350	Normal operation
12			< 300	0	0	< 300	0	6 years after implementation of the principles of proper operation
13			720	700	700	1400	0	Normal operation
14			310	< 300	0	1200	0	1 year after implementation of the principles of proper operation
15			300	< 300	0	1500	0	2 years after implementation of the principles of proper operation
16			< 300	0	0	390	0	3 years after implementation of the principles of proper operation
17			1370	1000	1000	8700	0	Operation, bad ACM conditions
18			390	350	400	1500	0	Operation limited to approximately 2/3 of all rooms in building
19			< 300	0	0	300	0	No operation for ~ 7 years
20			854	680	500	1500	< 300	8 months after asbestos removal
21			< 300	0	0	< 300	0	6 years after asbestos removal

^a^A long-wall structure with brick walls or frame structure, reinforced concrete, containing sandwich element with asbestos-cement plates.

^b^Lightweight steel construction with sandwich walls containing asbestos plates.

The median concentration of dust is also within the wide range of 0–800 (f/m^3^) depending on the model of use or technical condition.

### Long-term consequences

Although there are differences in the content and types of asbestos in individual products, the approximate content in typical and often used ACM are similar in many countries^19,20,29^. Therefore, conclusions of dust changes and the described building self-cleaning process applies to a broad range of building systems with similar characteristics. The OM method requires high determination precisions and maintaining high analyst proficiency in inter-laboratory tests^47,48^. Test results obtained in the past for the concentration of respirable fibers with ø > 0.2 µm in used buildings are now are out of date. Real values are often at or below the limit of OM quantification^49,50^. Many of the systems presented here ware characterized in a range from 0 to 300 f/m^3^. They will fulfil this condition for many years into the future without modification.

Regarding the process of changes in dust concentration during the operation of buildings, it can be assumed that buildings are subjected to opposing factors during their operation. The first group of factors includes: ageing of products, operational vibrations of products and ACM structures caused by the building environment, mechanical damage caused by renovation works, air movement in rooms, etc. They cause an increase in the concentration of dust in the air.

The opposite factors, such as air exchange (airing, ventilation), sedimentation of suspended dusts (increasing with time), cleaning procedures of the building (periodical and daily), introduction of additional insulation layers covering ACM products (painting, impregnation, separation by partitions e.g. with gypsum cardboard board of internal products or an additional layer insulating the building and covering external ACM products), they reduce concentration of dusts in indoor air. The combination of these two and the predominance of a specific group of factors will ultimately determine the emergence of a specific trend and a statistically significant tendency or lack thereof. This may become evident over time, but time, as the only factor, is not the leading one causing changes in fibre concentration in indoor air. Testing over a longer time period makes it possible to detect the predominance of certain factors in the form of a trend. In most of the cases analyzed there is a reduction in the fibre content of indoor air. Decrease of concentrations of asbestos fibres in indoor air have place if ACM not come into direct contact with the air inside the building. It leads to a reduction of this contaminant over time to outdoor air levels. This applies to buildings with non-friable ACM, without deterioration, and any vibration of the building and/or structure that may be transmitted to the ACM. An example of this construction is BISTYP.

The health aspect of asbestos works must be taken into account relative to contemporary tendencies leaning towards absolute asbestos removal. Pleural mesothelioma cancer is characterized by a 40-year latency period. Therefore, results of exposure from 1990 to 2019 will result in diseases arising in 2030–2059. This applies in particular to the higher asbestos exposures arising during the asbestos removal programs. Accelerated and imprecise disassembly, may result in increasing mesothelioma in future decades. Research on the increase in mortality in the USA in the 1980s caused by asbestos removal confirmed this problem.

## Conclusions

1. The concentration of asbestos fibers in indoor air of “rigid-constructions” is most often small, ~ 0–300 f/m^3^; “non-rigid construction” is often higher.
2. In a such buildings after many years of operation the air quality getting better over time.
3. Active behavior in buildings with asbestos is a cause of above-normal dust pollution. For this reason, children and young people should not use buildings with asbestos, regardless of their physical condition.
4. In a normally used building containing ACM, ventilation and air exchange are important factors in reducing the concentration of asbestos dust over time.
5. If there is no evidence of an increase in the concentration of asbestos in the air, the removal of ACM from such facilities should be postponed until the building is no longer used.
6. Repeated confirmation of low concentrations and/or confirmed concentration decreases in the building allows an extension of safe facility operation.
7. The reduction of asbestos dust in buildings can be a normal and natural process after proper and long service life (if the operation is not accompanied by the destruction of asbestos). Such conditions are met by many buildings with non-friable ACM, in which asbestos is insulated from the internal air. An example of this construction is BISTYP.

## Supplementary Information

Supplementary Information.

## Data Availability

The datasets generated during and/or analyzed during the current study are available from the corresponding author upon reasonable request.

## Abbreviations and definitions

ACM: Asbestos containing materials
a–c: Asbestos–cement (product, materials, etc.)
ARD: Asbestos-related diseases
CSRS: Czechoslovak Republic (*Československá socialistická republika*—*ČSSR*), was part of the Eastern Bloc
HEPA: High efficiency particulate air filter
GK: Gypsum plasterboard
MMMF: Man-made mineral fibres
NOAEL: No-observed adverse effect levels
GDR: German Democratic Republic (German Deutsche Demokratische Republik, DDR, colloquially East Germany)—a non-existent German state that was part of the Eastern Bloc
OM: Optical microscope
PLM: Polarized light microscope
PCM: Phase contrast microscope
PCOM: Phase contrast optical microscope
PEL (OEL): Permitted exposure limits (occupational exposure limits
PW3/A (and its modification, e.g. PŻW3/A/S): Sandwich panels used up to the 90 s in skeletal construction in layered curtain walls: thermal insulation core made of mineral wool or foamed polystyrene in a wooden frame, double-sided asbestos-cement flat plate. The panels were attached to the steel structure of curtain walls. From the inside, they were covered with plasterboards
“rigid” construction with asbestos: Building structures with masonry or reinforced concrete walls, containing sandwich walls with non-friable asbestos (e.g. PW3/A)
“non-rigid” construction with asbestos: Light steel frame structure of the building (e.g. building type LIPSK”) with sandwich walls with friable or/and non-friable asbestos
SEM–EDS: Scanning electron microscopy–energy dispersion spectroscopy
TEM: Transmission electron microscopy
“work area”: Closed, encapsulated space in the building where asbestos dismantling works are carried out
