# Supplementary material for: The natural reduction of threat in selected systems of old buildings containing asbestos
Source: Sci Rep. 2022 Feb 16;12:2580. doi: 10.1038/s41598-021-04487-y (PMC8850589; doi:10.1038/s41598-021-04487-y)
Supplement: Supplementary file 1 — Supplementary Information. [file 41598_2021_4487_MOESM1_ESM.docx]

**Table 1.1** Changes for Buildings 1, 2 and 3 in three time intervals

| Changes in Building 1  between 8 and 36 weeks | Changes in Buildings 2 and 3 in  Period of the first 10 weeks after the start of the study | | Changes in Buildings 2 and 3 during  between 24 and 48th week after the start of the study | | Changes in Buildings 2 and 3 in the whole interval of study from 0 to 48th week | |
| --- | --- | --- | --- | --- | --- | --- |
| Number of weeks/fibre concentration in buildings | | | | | | |
| Building 1 | Building 2 | Building 3 | Building 2 | Building 3 | Building 2 | Building 3 |
| 8/740  24/850  24/1500  36/1700  36/2200  36/3500 | 2/800  3/500  4/600  5/900  6/1200  7/1500  8/800  10/700 | 0,5/1000  2/760  3/800  4/330  5/600  6/1350  6/220  7/440  8/740  10/600 | 24/350  36/550  48/800 | 24/570  36/800  36/250  36/500  48/420  48/190 | 2/800  3/500  4/600  5/900  6/1200  7/1500  8/800  10/700  24/350  36/550  48/800 | 0,5/1000  2/760  3/800  4/330  5/600  6/1350  6/220  7/440  8/740  10/600  24/570  36/800  36/250  36/500  48/420  48/190 |

Table 1.2 Determination of Pearson correlation

| Buildings 1, 2, 3 in selected time periods | Pearson correlation coefficient r | Coefficient of determination R^2^ | p>  0,1 | p | Interpretation |
| --- | --- | --- | --- | --- | --- |
| Building 1 between the 8th and 35th week of the study | 0,742 | 0,55 |  | 0,092 | strong correlation , statistical tendency |
| Building 2 between the 2nd and 10th week of the study | 0,281 | 0,08 |  | 0,500 | low correlation, insignificant association, no statistical tendency |
| Building 2, tested between 24-48 weeks | 0,999 | 1 |  | 0,033 | very strong correlation, statistically significant association |
| Building 2 throughout the test interval | -0,274 | 0,08 |  | 0,415 | low correlation, insignificant association, no statistical tendency |
| Building 3 tested up to 10 weeks | -0,243 | 0,06 |  | 0,500 | low correlation, insignificant association, no statistical tendency |
| Building 3 between 24-48th week | -0,495 | 0,25 |  | 0,318 | moderate correlation, insignificant association, no statistical tendency |
| Building 3 - throughout the test interval | -0,439 | 0.19 |  | 0,089 | moderate correlation, statistical tendency |

Note:

p *–* the *p* - value of the test (*test probability)*

p < 0,05 the relationship between elapsed time between tests and dust is statistically significant

0,05 < *p* < 0,1 this relationship is significant at the level of statistical tendency

*r* - correlation coefficient*,* the estimated measure of association corresponding to the method employed

Strength of correlation coefficient value |r|

|  |  |
| --- | --- |
| 0-0,3 | low correlation |
| 0,3-0,5 | moderate correlation |
| 0,5-0,7 | strong correlation |
| 0,7-1 | very strong correlation |

Graphical presentation of the analyses: In Figs. 1 to Fig. 7, the vertical axis is the fibre concentration [f/m^3^], the horizontal axis represents the time lapse in weeks.


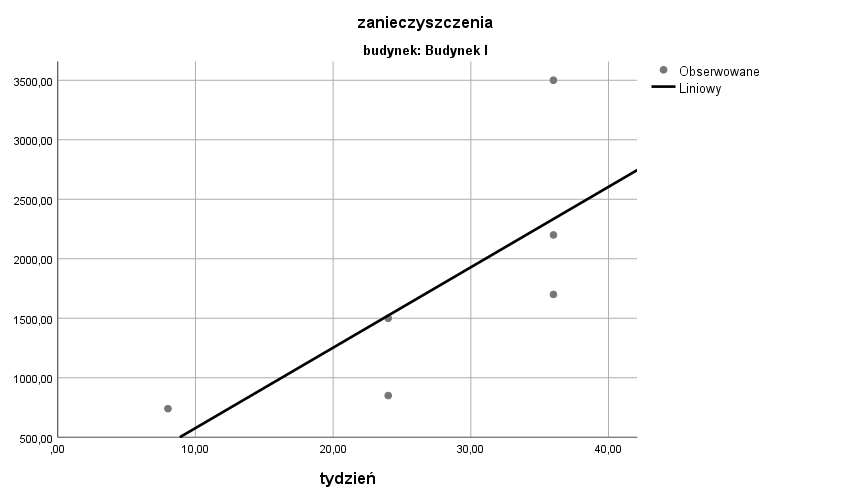


Fig. 1 Building 1 from week 10 – 40


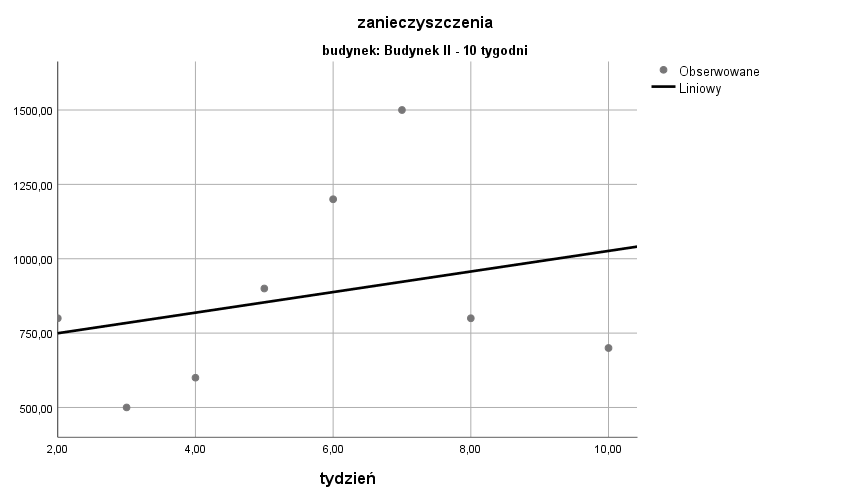


Fig. 2 Building 2 in study: up to week 10


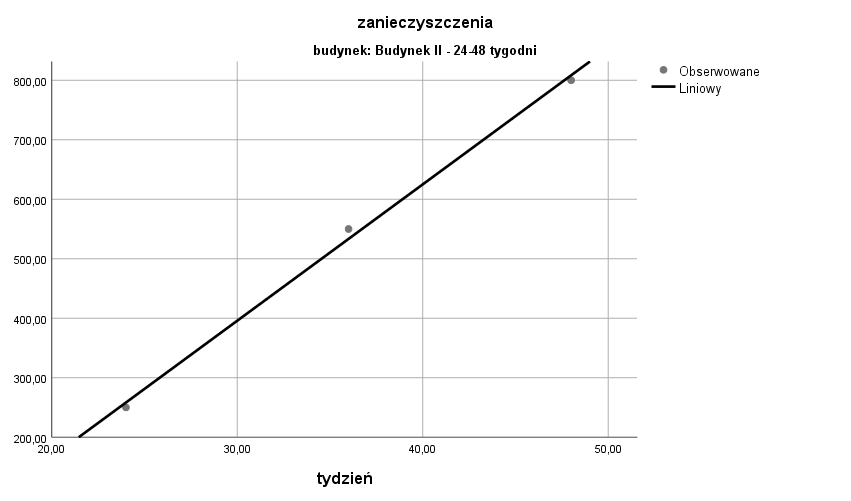


Fig. 3 Building 2 in survey from week 20 to 48


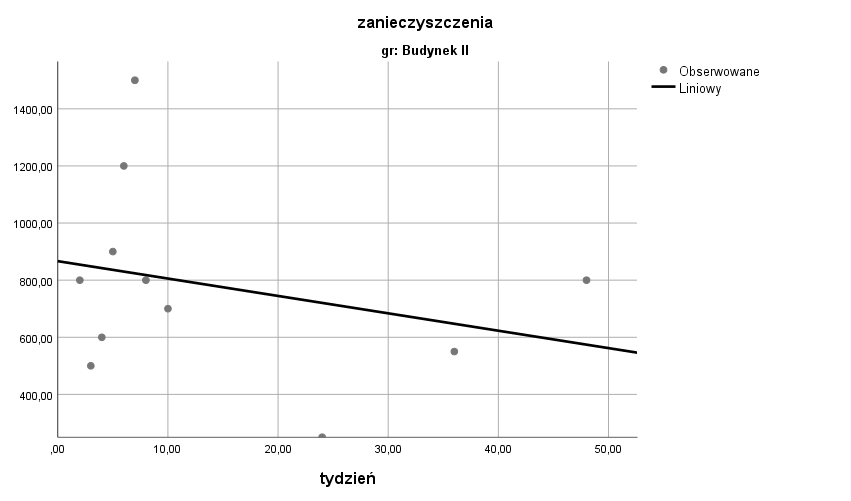


Fig. 4 Building 2 throughout the study interval, from week 0 to week 48


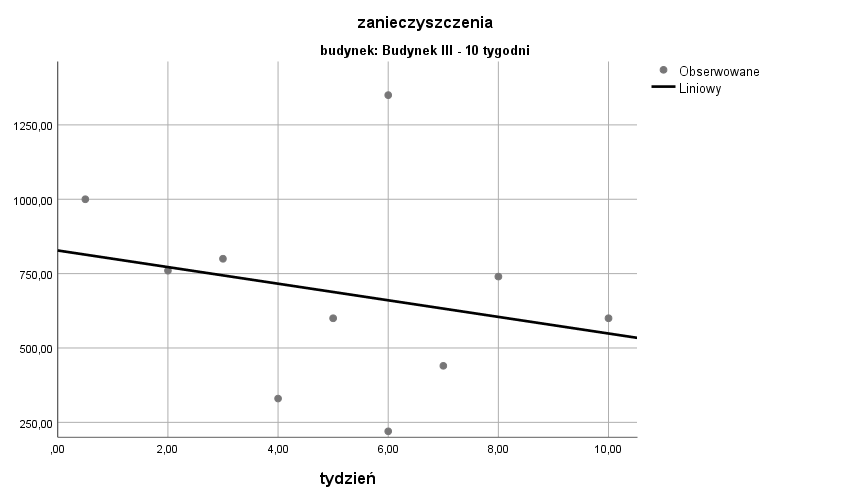


Fig. 5 Building 3, contamination during the first 10 weeks of the study


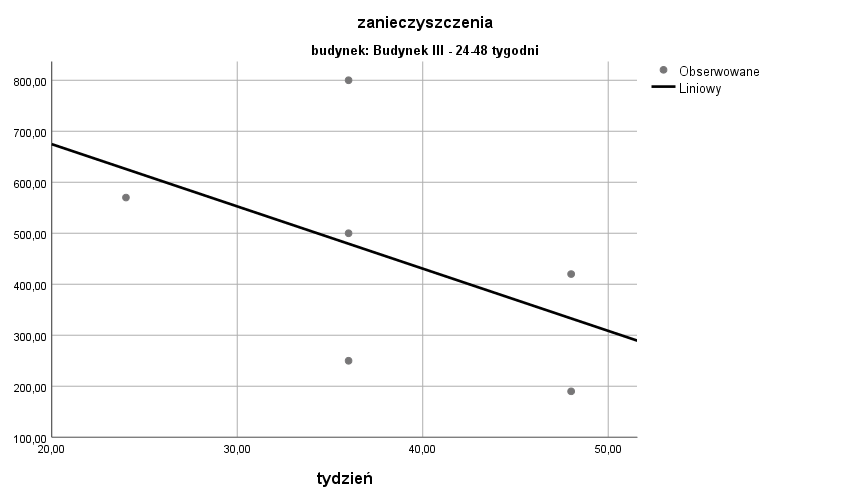


Fig.6 Building 3 from week 20 to week 48


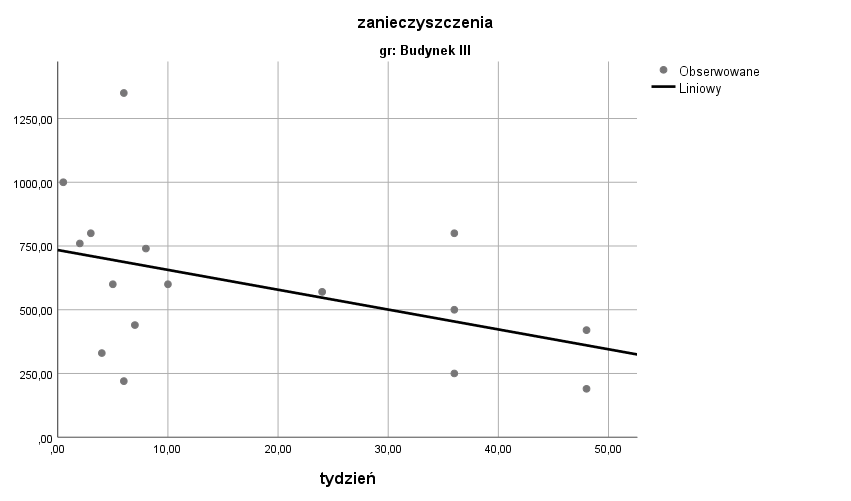


Fig.7 Building 3 throughout the test interval, from week 0 to week 48

**Interpretation of results**

The data presented means that for both buildings, changes in asbestos concentrations in the air over time can be explained in 8% of the results for building 2 and in 19% of the results for building 3.

- Building 2, is characterized by a low correlation of the studied features (concentration/time) (temporarily, over a short time it can be strong and positive). The correlation of the features at the statistical level is insignificant, there is no statistical tendency of the changes throughout the study period of 48 weeks.
- Building 3 is feature correlation (concentration/time) - negative, moderate; feature correlation at the level of statistical tendency.

The study detected two associations of the studied traits at the level of statistical tendency - for objects:

- No. 1 - between 8 and 36 weeks. It is a strong positive correlation. It indicates an increase in dust concentration with the passage of time. This is due to the drying of the walls after asbestos removal, during which the wet cleaning technique was used. This was carried out inaccurately. This resulted in the release of residual unremoved asbestos fibres from the wet walls into the environment and an increase in indoor air pollution, despite the absence of ACM products.

- No. 3, over the entire time interval from week 0 to week 48 .

This is a situation where a decrease in dust concentration is not apparent in the short term, but it is drawn as a tendency over a longer period of time.

Statistical analysis over the whole study period showed a low to moderate linear correlation between the passage of time and the decrease in dust concentration. For Building 3 this may give a statistical trend of this decrease. It is to be expected that in building 3, the introduced concentration-reducing factors may have a temporal effect on the concentration decrease if they dominate the factors causing the concentration increase over a longer period of time. The investigated duration of this dominance for building 3 was short so the correlation is weak and the change in concentration is a statistical tendency.
